# Homology Modeling and Optimized Expression of Truncated IK Protein, tIK, as an Anti-Inflammatory Peptide

**DOI:** 10.3390/molecules25194358

**Published:** 2020-09-23

**Authors:** Yuyoung Song, Minseon Kim, Yongae Kim

**Affiliations:** Department of Chemistry, Hankuk University of Foreign Studies, Yongin 17035, Korea; yysong1996@naver.com (Y.S.); alstjs032@naver.com (M.K.)

**Keywords:** autoimmune diseases, rheumatoid arthritis, truncated-IK protein (tIK protein), anti-inflammatory peptide, interleukin-10, homology modeling, expression

## Abstract

Rheumatoid arthritis, caused by abnormalities in the autoimmune system, affects about 1% of the population. Rheumatoid arthritis does not yet have a proper treatment, and current treatment has various side effects. Therefore, there is a need for a therapeutic agent that can effectively treat rheumatoid arthritis without side effects. Recently, research on pharmaceutical drugs based on peptides has been actively conducted to reduce negative effects. Because peptide drugs are bio-friendly and bio-specific, they are characterized by no side effects. Truncated-IK (tIK) protein, a fragment of IK protein, has anti-inflammatory effects, including anti-rheumatoid arthritis activity. This study focused on the fact that tIK protein phosphorylates the interleukin 10 receptor. Through homology modeling with interleukin 10, short tIK epitopes were proposed to find the essential region of the sequence for anti-inflammatory activity. T_H_17 differentiation experiments were also performed with the proposed epitope. A peptide composed of 18 amino acids with an anti-inflammatory effect was named tIK-18mer. Additionally, a tIK 9-mer and a 14-mer were also found. The procedure for the experimental expression of the proposed tIK series (9-mer, 14-mer, and 18-mer) using bacterial strain is discussed.

## 1. Introduction

Inflammation is a protective response to harmful stimuli in the human body, involving immune cells, blood vessels, and inflammatory mediators [1]. About 30% of the diseases in humans are known to be related to inflammation. In particular, various diseases caused by chronic inflammation, such as diabetes, cardiovascular disease, high blood pressure, and arthritis, are very difficult to treat, and some of the exact pathophysiologies are unknown [2]. Currently, these diseases of unknown etiology can be treated with anti-inflammatory drugs. Anti-inflammatory drugs are generally classified into steroidal anti-inflammatory drugs and nonsteroidal anti-inflammatory drugs (NSAIDs) [3].

The steroidal anti-inflammatory drugs currently used are associated with a variety of side effects, such as gastrointestinal disorders, gastric ulcers, kidney diseases, and bleeding [4]. Even nonsteroidal anti-inflammatory drugs used to reduce the side effects of these inflammatory diseases can also affect the stomach and kidneys negatively [5]. Over the past decade, biopharmaceutical research has focused on peptide drugs to reduce these negative effects. Peptide drugs are preferable alternatives to small molecules and biological therapeutics because of their biological characteristics. Peptides are differentiated by their bio-friendly and bio-specific nature, their most remarkable advantages. In other words, there are few side effects and even a small dose exhibits strong pharmacological activity [6,7].

Arthritis is a term that usually refers to joint pain in one or more joint junctions, and all types caused by excessive immune system attacks are called inflammatory arthritis [8]. A representative disorder is rheumatoid arthritis, a subtype of autoimmune diseases that results from inflammation of the immune system due to genetic factors and environmental influences [9]. One of the causes of rheumatoid arthritis is the imbalance of cytokines [10]. Cytokines are protein immune-modulators secreted from immune cells and play crucial roles in the expression and persistence of rheumatoid arthritis. These can be categorized as pro-inflammatory cytokines that activate and maintain inflammation and anti-inflammatory cytokines that inhibit or regulate inflammation [11]. In other words, the disease occurs when the activity of pro-inflammatory cytokines is greater than that of the anti-inflammatory factors. Therefore, this suggests that a substance that can balance cytokines may be a candidate for the treatment of rheumatoid arthritis.

In a recent study, inhibitor K562 (IK) protein was firstly isolated from a K562 leukemia cell line in culture [12]. It is an inhibitory regulator of inflammatory cytokine expression and is known as a new anti-inflammatory candidate that adjusts the imbalance between pro-inflammatory and anti-inflammatory factors [13]. IK protein inhibits the expression of MHC (major histocompatibility complex) II induced by IFN-γ. In the full-length IK cytokine, the protein obtained by translating only from the 316 methionine position to 557 tyrosine is called the truncated IK (tIK) cytokine. This protein has also been shown to downregulate inflammatory cytokines to a level similar to full-length IK protein [13]. It can be seen in the inflammatory arthritis mouse model that tIK cytokines reduce arthritis symptoms. These results indicate that tIK cytokine may function to partially prevent the induction of inflammatory cytokines and thus, mitigate the progression of joint inflammation and damage in rheumatoid arthritis [13]. It also demonstrated that when tIK protein was expressed, various signaling proteins were phosphorylated. In other words, tIK protein interacted with these proteins, resulting in an anti-inflammatory effect.

However, the three-dimensional structure of tIK is not known, and the molecular weight of tIK (29 kDa) is too large to be developed as pharmaceutical products. This can also cause problems in manufacturing cost, time stability, etc. when produced in large quantities. Therefore, the study was conducted to propose a short length epitope including an essential part of the tIK structure. The 3D structure of tIK was constructed through homology modeling with MSA (Multiple Sequence Alignment) using computational simulation. Firstly, their homologous proteins were searched by PSI-BLAST (Position-Specific Iterative Basic Local Alignment Search Tool). PSI BLAST produces a position-specific scoring matrix (PSSM) from multiple sequence alignments of sequences with a score above the threshold using a protein-protein sequence similarity search tool. The next step is MSA, which finds whether there is a protein with a similar structure in each part of the full-length protein. When revealing the initial structure of most proteins, there is nothing to contrast with full-length, so MSA can predict the structure of the partial part.

Among the various signaling proteins of the arthritis mouse model, we focused on the phosphorylation of tyrosine 496 in interleukin 10 receptor subunit alpha (IL-10Rα). IL-10 is a prototype of anti-inflammatory cytokines and is known to be an important factor in inhibiting inflammatory cytokines. In addition, IL-10 inhibits the cytokines IL-1, IL-6, TNF-α, and IFN-γ, which are frequently produced in rheumatoid arthritis sites [14]. IL-10 binds to the extracellular domain of the IL-10R and forms a homodimeric form. Ligand binding causes JAK1 and TYK2 to bind to the intracellular domain of the IL-10R and activate the signaling pathway. Subsequently, phosphorylation of tyrosine residues occurs at Y446 and Y496 by JAK1 and TYK2, and STAT3 (signal transducer and activator of transcription 3) binding occurs by phosphorylation. This results in the inhibition of pro-inflammatory cytokines [15]. We expected that IL-10 and tIK proteins would act on IL-10 receptors with similar structure and function with anti-inflammatory effects [16]. Sequence and structural similarity between tIK protein and IL-10 were confirmed through MSA and homology modeling. Based on these results, epitopes were derived based on the fact that the active site of tIK is similar to the binding site of IL-10.

In this study, tIK epitope was proposed to predict the three-dimensional structure of tIK through PSI-BLAST and MSA, and to find the amino acid sequence exhibiting the anti-inflammatory activity of tIK. A TH17 differentiation experiment with tIK epitope was performed to derive tIK-18mer showing anti-inflammatory effect, and tIK-9mer and tIK-14mer having shorter sequences than 18mer were also found. Since a large amount of protein is required to study the three-dimensional structure of the three types of tIK series (tIK-9mer, tIK-14mer, and tIK-18mer), experiments were conducted using bacterial strains for large-quantity production. The process of optimizing the expression and isolation and purification process of the tIK series will be discussed in this report.

## 2. Results and Discussion

### 2.1. Proposal of tIK Epitope Sequence with Anti-Inflammatory Effect

**Figure 1:**
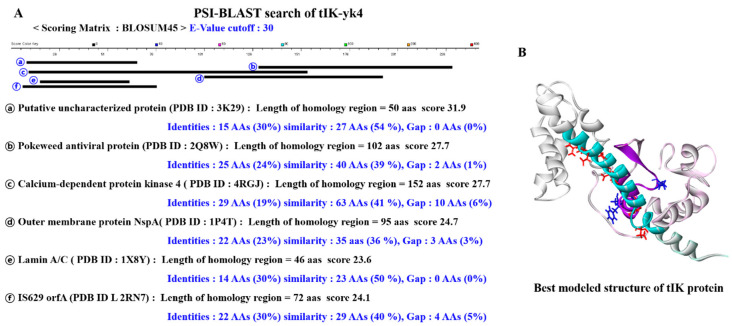
3D structure of tIK by computational simulation. (**A**) When performing with the Position-Specific Iterative Basic Local Alignment Search Tool (PSI-BLAST), BLOSUM45 was used as the scoring matrix and the E-value cutoff was 30. Based on the high scores, six highly homologous proteins were identified and these templates are represented from ⓐ to ⓕ. Of the six templates, ⓑ, ⓒ, and ⓕ that can encompass the full sequence of tIK were selected, and the 3D structure of the tIK protein obtained from this is shown in (**B**).

For epitope-based peptide drug designs, a 3D structure of the tIK protein was constructed using a computational simulation technique by using Discovery Studio 4.5, which provides molecular modeling solutions for bio and drug development. The tIK sequence was input into PSI BLAST to find proteins with similar sequence alignment that can be used to find the expected structure for each part of the full-length tIK. Since the results of the PSI BLAST search did not reveal high homology proteins for the entire sequence of tIK, several homologous proteins were selected and MSA was performed using them (Figure 1A, Figure S1). The sequence alignment result produced by MSA and the 3D structure of the template proteins were applied to homology modeling and the 3D structure of the tIK was constructed through a model refinement process. Finally, the structure obtained through energy minimization is shown in Figure 1B.

**Figure 2:**
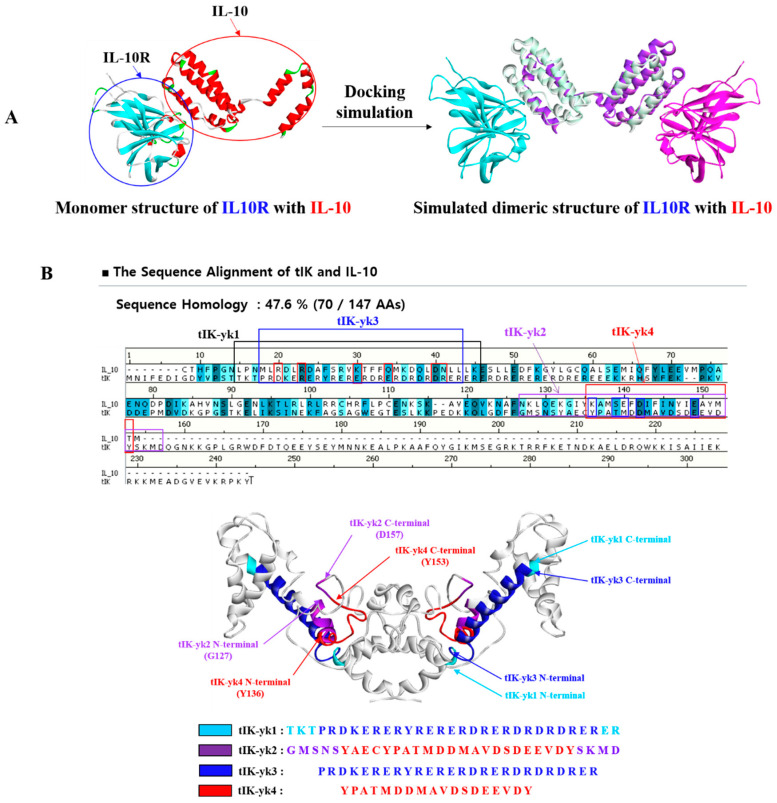
(**A**) Through docking simulation, it was visually confirmed that the IL-10R and IL-10, which are phosphorylated by the tIK protein, bind in homodimeric form. The left part of the Figure shows the monomeric structure of IL-10R and IL-10. (**B**) Sequence alignment of tIK and IL-10. Considering that tIK protein phosphorylates IL-10R, homology modeling was performed with the full-length sequence of the IL-10 and tIK proteins. The degree of homology was about 48%, indicating very high sequence similarity between the two peptides. The higher the homology for each amino acid portion, the darker the color of the corresponding area. IL-10 is bound to the IL-10R, indicated by a rectangular box. Four tIK epitope sequences were proposed based on an active site that is highly homologous to IL-10.

In the tIK transgenic mouse, the pattern of phosphorylation changes in various receptors and intracellular proteins was analyzed, which showed a large change in IL-10Rα. Activation of IL-10R also induced tyrosine phosphorylation of JAK1 and TYK2 kinases, which was consistent with the phosphorylation of tyrosine 496 identified. Since the function of the protein is closely related to the tertiary structure, IL-10 and IL-10R form a homodimeric structure and bind to each other, which was confirmed through PDB and docking simulation (Figure 2A). Based on the fact that tIK protein caused the phosphorylation of IL-10Rα in transgenic mouse experiments, it was expected that IL-10 and tIK proteins would act on the IL-10 receptor with similar functions and anti-inflammatory effects. Sequence and structural similarity between tIK protein and IL-10 were confirmed by homology modeling (Figure 2B). In general, the structure is similar when there is more than 30% homology [17]. A comparison of the two sequences confirmed a high homology of about 48%. The homology modeling of tIK and IL-10 showed high homology with tIK protein, especially in the region where IL-10 binds to IL-10R. This suggests that tIK protein, which has high homology with IL-10, will also interact with IL-10R with similar homodimeric structure (Figure S2). However, as mentioned earlier, the full length of the tIK protein itself is too long to use as a medicine because it consists of 242 amino acids. For this reason, epitopes were screened with superior anti-inflammatory activity and shorter amino acid sequences than the full-length tIK. Therefore, tIK epitopes with a total of four short peptide sequences (tIK-YK1, 2, 3, and 4) were proposed by selecting epitopes comprising the tIK regions that were highly homologous to the active site of IL-10.

**Figure 3:**
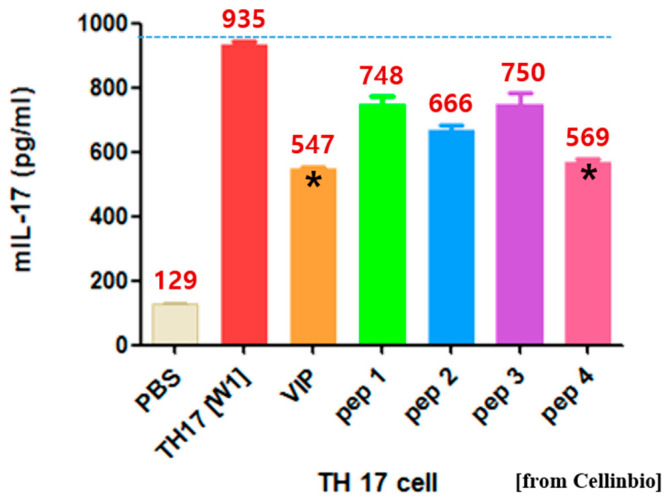
Anti-inflammatory effect of the four peptide candidates in a T_H_17 cell differentiation inhibition model. The results show that tIK-yk4 (pep 4), composed of 18 amino acids, shows an anti-inflammatory effect comparable to vasoactive intestinal peptide (VIP). Both are marked with *.

T_H_17 cell differentiation experiments were performed to confirm the anti-inflammatory effect of the four proposed tIK epitopes. Recently, it has been reported that IL-17, released from T_H_17 cells, a new cell population, is important in autoimmune diseases [18]. T_H_17 cells are differentiated by IL-23, which has been shown to increase the expression of IL-17 and play an important role in the development and activity of rheumatoid arthritis [19]. In addition, a commercially available vasoactive intestinal peptide (VIP) was used as a control to demonstrate the anti-inflammatory effect of tIK. VIP is a powerful anti-inflammatory agent that regulates homeostasis of the intestinal epithelial barrier [20]. The results of the anti-inflammatory test of the four epitopes showed that the peptide with the best anti-inflammatory activity was tIK-YK4 (Figure 3). This was an epitope consisting of 18 amino acids that was later named tIK 18-mer. Shorter derivatives 14-mer and 9-mer were also proposed through the structure of the 18-mer. Their anti-inflammatory effects were also tested and 9-mer showed similar efficacy compared to 18-mer (Figure S3). Thus, confirming their potential as a new anti-inflammatory candidate.

### 2.2. Construction of Expression Vectors

**Figure 4:**
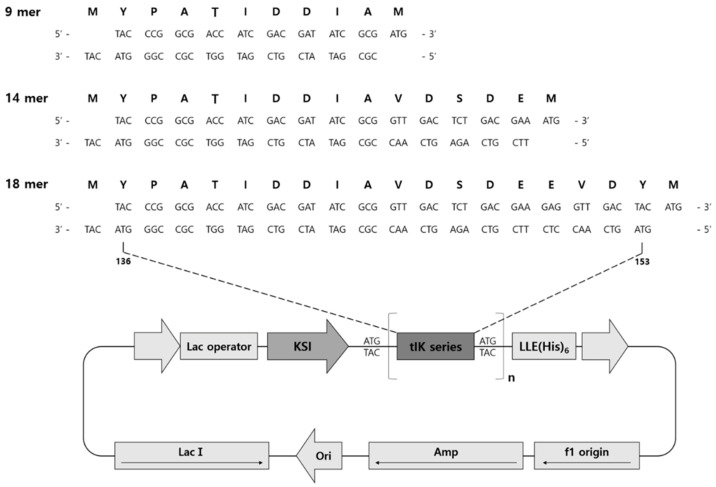
Schematic representation of the ketosteroid isomerase (KSI)-fused expression vector for the tIK series (9-mer, 14-mer, 18-mer). A commercial vector, pET31b (+), was used, and the DNA and amino acid sequences of the inserts are shown on the vector map. The tIK series was expressed as a fusion protein consisting of KSI (ketosteroid isomerase) to make it hydrophobic and a His_6_-tag was added for purification.

Single-stranded tIK series were made double-stranded with complementary DNA for application to the pET31b (+) vector (Figure 4). An annealing process was performed in which two single strands of DNA were spontaneously bound to the complementary strands. To confirm that the annealing process worked well, the DNA was separated by electrophoresis on a 2% agarose gel. The non-annealed strands were so small that they ran through the gel without forming a band, so only the well-stranded double helix DNA was screened. Double-stranded DNA was extracted from the gel and these target peptide-coding sequences were ligated into a pET31b (+) vector to obtain plasmids. To express the pET31b (+) vector into which the DNA double-strands were inserted, it was transformed into competent cells. After the procedure, the product was incubated overnight on LB agar plates containing carbenicillin. The plasmid was removed from the competent cells to determine how many DNAs were inserted into the vector during ligation. After plasmid purification and restriction digestion, the number of inserts in the 1.5% agarose gel was determined. Final constructs for the expression of the tIK series had a ketosteroid isomerase (KSI) fusion partner at the N-terminal and a His_6_-tag and CNBr cleavage site at the C-terminal. Isopropyl β-D-1-thiogalactopyranoside (IPTG) induction experiments were used to identify the host strains to be used to express the fusion proteins. The expression levels of the fusion proteins from the three expression hosts were confirmed by the band density of the expressed proteins.

### 2.3. Expression of tIK-9mer, 14mer, 18mer

**Figure 5:**
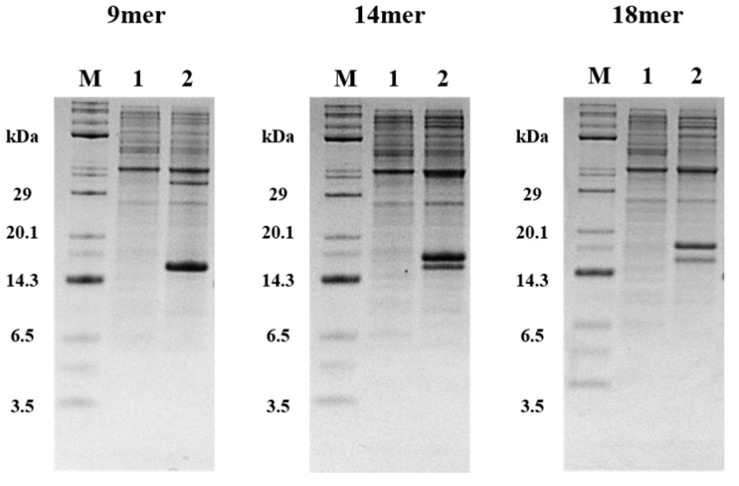
Tris-tricine 12% sodium dodecyl sulfate- polyacrylamide gel electrophoresis S (DS-PAGE) diagrams showing the expression of the tIK series. Lane M—molecular weight marker; lane 1—cells before IPTG induction; lane 2—cells after IPTG induction showing a KSI-fused protein band. The expressed tIK series is insoluble due to KSI and thus, remains in pellet form. Therefore, it can be seen that bands are formed only in lane 2 of each gel.

In the host cell expression test, C41(DE3) was selected for the tIK-9mer and C43(DE3) was selected for the tIK-14mer and tIK-18mer. Unlabeled tIK series were successfully obtained by growing bacteria in M9 minimal media as described above. Figure 5 shows the tris-tricine SDS PAGE analysis of the expression of the KSI-tIK series-His_6_ tag. The addition of 1 mM IPTG to the growth medium allowed for over-expression of the tIK series. Since KSI was used as a fusion partner, the fusion protein can be expressed as an insoluble protein. Finally, the peptides were obtained in the form of pellets by centrifugation.

In this study, an epitope-based anti-inflammatory peptide, tIK, was predicted by computational simulations, including PSI BLAST, docking simulation, and homology modeling, followed by a T_H_17 cell differentiation test, which indicated anti-inflammatory activity. After demonstrating the possibility as a pharmaceutical drug candidate, vector selection and expression of each peptide differing in length of amino acid sequences were carried out for large-quantity production. The final aim of our study was the structural identification of tIK showing positive effects on autoimmune disease and further demonstrating the possibility of truncated interleukin peptides as peptide drugs with no adverse effects, in contrast to low-molecular medicine. Protein function is closely related to tertiary structure, both of which play their respective roles in the living body. Therefore, subsequent studies will focus on the expression, purification, and identification for investigating the function of truncated peptides. In order to demonstrate the anti-inflammatory activity more accurately, we plan to confirm the suppression of inflammatory cytokines that appear when treatment with tIK peptide, which removes LPS, which appears when proteins are expressed in *Escherichia coli* after successful purification. In addition, for protein structure studies through NMR spectroscopy, we are trying to optimize the process of expressing the uniformly/selectively ^15^N labeled tIK peptide. Because of the promising strength derived from its inherent membrane-penetrating ability, selectivity, and specificity, we expect to find new medicine for incurable diseases by studying the peptide drug candidates.

## 3. Materials and Methods

### 3.1. D Structure Prediction of tIK and Selection of tIK Epi Escherichia coli Tope

Constructing and validating the modeled structures was performed by through PSI BLAST, MSA, homology modeling, and refinement processes, which are computational simulations. PSI BLAST was used to find proteins homologous to the tIK protein (Figure 1A). Proteins homologous to tIK protein were searched using a BLOSUM45 scoring matrix with an E-value (Expect-value) cutoff of 30. The E-value is a parameter that describes the number of hits one can expect to see by chance when searching a database of a particular size. MSA of these proteins was performed to find similar structures for each part of the tIK protein, and homology modeling was performed to compare multiple template proteins with the tIK protein. The sequences of tIK protein were aligned to template proteins: 2Q8W, 4RGJ, and 2RN7. MSA results were scored using BLOSUM45 scoring matrix with an E-value (Expect-value) cutoff of 30. Finally, the results were obtained by the precise refinement of the structure through energy minimization (Figure 1B). The position and orientation of tIK protein were optimized by using verified protein (3D-profile) for structure selection, which is validation of protein structure that is built by using homology modeling. Discovery Studio 4.5 (Biovia, San Diego, CA, USA) was used [21,22,23,24].

Protein function is closely related to its tertiary structure, therefore, the binding of IL-10R and IL-10, which delivers signals for anti-inflammatory activity, was confirmed. The structures of the extracellular domain monomer of IL-10Rα (PDB ID: 1Y6K_R) and IL-10 monomer (PDB ID: 1Y6K_L) were obtained from the Protein Data Bank (PDB). Since IL-10 and IL-10R are homodimers, the docking protocol was used to confirm the formation of the dimers (Figure 2A). At this time, the distance cutoff in the filter pose was 10.0, and the angular step size, which means step size of rotation for ligand protein during docking stage, was 6. Additionally, ZRank was set to True, top poses were 2000, and report score components were false. Clustering representing docking models contained the top poses, top poses were 2000, RMSD cutoff was 10.0, interface cutoff was 10.0, and maximum number of clusters was 100.

Because the greatest change in the phosphorylation pattern of IL-10Rα was due to the tIK protein, we compared the structure of tIK protein and IL-10 (Figure 2). Comparing the sequences of the two proteins revealed a high degree of homology of about 48%, particularly in areas where IL-10 binds to the IL-10R. Since IL-10 is a homodimeric structure, tIK also showed a very similar structure when expressed in homodimeric form in the monomer. Because the binding regions of IL-10 and IL-10R in the tIK protein sequence were expected to be the major reason for anti-inflammatory activity, four different tIK epitopes were proposed based on the binding site.

### 3.2. T_H_17 Cell Differentiation

T_H_17 cell differentiation was performed by Cellinbio to determine the highest anti-inflammatory activity in the group of four epitopes (Figure 3). According to the manual, Naïve CD4+ T cells were isolated from the spleen of WT Balb/c mice with a magnetic-activated cell sorting (MACS) CD4+ T Cell Isolation kit (Miltenyi Biotec, Cologne, Germany). The cells were cultured for two days in medium, and then the mIL-17 concentration in 100 μL of the supernatant was analyzed using mIL-17 ELISA (enzyme-linked immunosorbent assay). Vasoactive intestinal peptide (VIP), which reduces inflammation and autoimmune components of rheumatoid arthritis, was used as a control. VIP indicated inflammation suppression contrary to the effect of T_H_17. Among the epitopes, the highest anti-inflammatory activity was found for peptides consisting of 18 amino acids, named the tIK 18-mer. Subsequently, testing was performed on a sequence obtained by further cutting the 18-mer. This test was repeated three times independently and the results are reported as the mean ± standard deviation.

### 3.3. Vector Construction

The base oligonucleotide sequences for the tIK-9mer, tIK-14mer, and tIK-18mer were chemically synthesized by Integrated DNA Technologies (Coralville, IA, USA). The two methionine residues present at the 5 and 8 sites in tIK peptide sequences were replaced with isoleucine to avoid cleavage (M5I, M8I; ATGATC). The synthetic oligonucleotide sequences and resulting amino acid sequences are shown in Figure 4. The forward and reverse primers were annealed by heating to 95 °C and cooling over 30 min at room temperature. After annealing, the cohesive ends were compatible with AlwN I. The annealed DNAs were isolated by 2% agarose gel and then purified by a DNA extraction kit (Qiagen, Germany) according to the manufacturer’s instructions [25,26]. The annealed DNA was ligated into an AlwN I-digested pET31b (+) vector (Novagen, Billerica, MA, USA). After that, to check insertion of the tIK gene in the vector, the ligated vector was transformed into Novablue (Novagen) competent cells and then plasmid DNA from the individual colonies was purified using a QIAprep Miniprep kit (QIAGEN) and digested with XbaI and XhoI (New England Biolabs, Ipswich, MA, USA). To obtain high yields of fusion proteins, recombinant plasmids with single or multiple inserts were transformed into various *Escherichia coli* (*E. coli*) strains, BL21(DE3) pLysS, BLR (DE3) pLysS (Novagen), C41(DE3), and C43 (DE3) (Avidis, France).

### 3.4. Expression of KSI-(tIK series)_n_-His_6_ Tag Fusion Protein

Various *E. coli* strains containing pET31b/KSI-(tIK sequence)-His_6_ tag were selected according to the expression level of the fusion protein (C41(DE3) for tIK 9mer and C43(DE3) for tIK-14mer and tIK-18mer) and plated on LB agar media. After overnight incubation, a 50 mL starter culture was inoculated from a single colony on an LB agar plate and grown in LB media overnight at 37 °C with shaking (250 rpm). The fully-grown culture (10 mL) was transferred to 1 L of M9 minimal medium and 1 g/L ammonium sulfate was added to M9 minimal medium. After incubation at 37 °C for 2–3 h, IPTG (1 mM) was added to the M9 minimal medium when the OD_600_ value was 0.5. The M9 minimal medium was incubated for 16–18 h and after that, the cells were harvested by centrifugation and stored at −80 °C for three hours or more. Expression of the tIK peptides were identified by 12% tris-tricine SDS-PAGE, as shown in Figure 5.

## 4. Conclusions

We used a computational simulation technique of Discovery Studio 4.5 to present a 3D structure of truncated-IK (tIK) protein that was not previously known. In the tIK transgenic mouse experiment, IL-10R and tIK were expected to function similarly because the phosphorylation pattern of IL-10Rα changed most significantly. IL-10 and tIK protein showed high homology of about 48%, especially at the site where IL-10 binds to IL-10R, an active site. Based on these homology modeling results, four epitopes were proposed for the first time based on the essential part of the tIK protein having anti-inflammatory activity. T_H_17 cell differentiation experiments with the proposed four epitopes confirmed that tIK-yk4 had anti-inflammatory effects and was named the tIK 18-mer, consisting of 18 amino acids. Based on this sequence, two other epitopes named tIK 9-mer and 14-mer were identified, which consisted of 9 and 14 amino acids, respectively.

In summary, the motif region of tIK, similar to the IL-10 active site, was selected as the tIK fragmentation candidate for the design of anti-inflammatory peptide drugs. Since there is no adequate inflammatory treatment with no side effects, studies of peptides as anti-inflammatory drugs are important. New pharmaceutical drug candidates can select the smallest unit with excellent physiological activity present in proteins to regulate biological signal transduction and function and can exhibit potent pharmacological action and activity, even in small amounts. The use of a peptide selected as the most useful or the most efficient in the treatment and prevention among specific proteins has the advantage of easy manufacturing, which can reduce the cost and the frequency of administration to the patient.

The appropriate competent cells for the expression of each of the tIK series proposed as candidate anti-inflammatory agents were identified and confirmed by agarose gel electrophoresis. The expression, purification, and analysis of peptides are generally difficult, but the expression process in this study was optimized. Furthermore, research is underway to secure peptides of high purity and high efficiency through the purification of peptides and study of the structure of the tIK series and the mechanism of action of anti-inflammatory peptides through NMR spectroscopy.

## Supplementary Materials

The following are available online.

## Abbreviations

IK	Inhibitor K562
tIK	truncated-IK
IL	Interleukin
IL-10Rα	Interleukin 10 receptor subunit alpha
TH17	T helper 17
MHC II	Major histocompatibility complex class II
IFN-γ	Interferon-γ
GPCR	G protein-coupled receptor
CVB3	Coxsackievirus
JAK1	Janus Kinase 1
TYK2	Tyrosine Kinase 2
STAT3	Signal transducer and activator of transcription 3
TNF-α	Tumor Necrosis Factor-α
PSI BLAST	Position-Specific Iterative Basic Local Alignment Search Tool
MSA	Multiple Sequence Alignment
E-value	Expect-value
IPTG	Isopropyl β-d-1-thiogalactopyranoside
OD600	Optical density 600 nm
SDS-PAGE	sodium dodecyl sulfate- Polyacrylamide gel electrophoresis
PSSM	Position-Specific Scoring Matrix
LB	Luria-Bertani
KSI	Ketosteroid isomerase
