# Supplementary material for: Homology Modeling and Optimized Expression of Truncated IK Protein, tIK, as an Anti-Inflammatory Peptide
Source: Molecules. 2020 Sep 23;25(19):4358. doi: 10.3390/molecules25194358 (PMC7583991; doi:10.3390/molecules25194358)
Supplement: Supplementary file 1 [file molecules-25-04358-s001.pdf]

Homology modeling and optimized expression of truncated IK protein, tIK, as an anti-inflammatory peptide

Yuyoung Song, Minseon Kim and Yongae Kim *

Supplementary

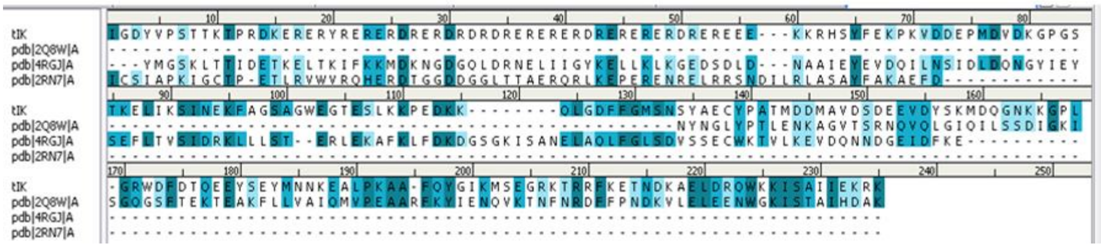

Supplementary 1. MSA and homology modeling using three selected template proteins and tIK.

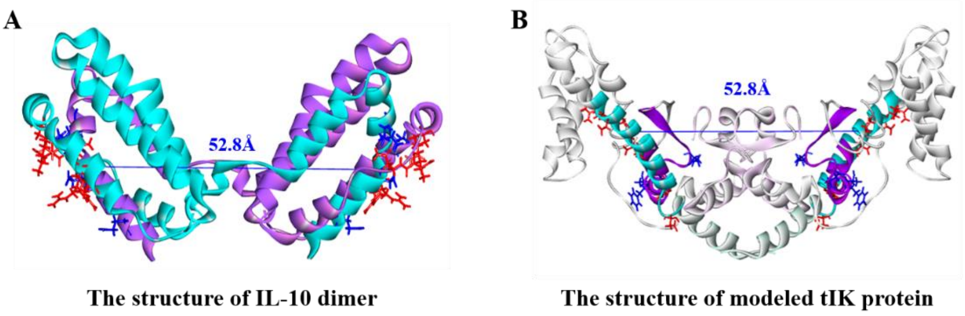

Supplementary 2. Comparison of IL-10 dimer and tIK dimer structures with high homology.

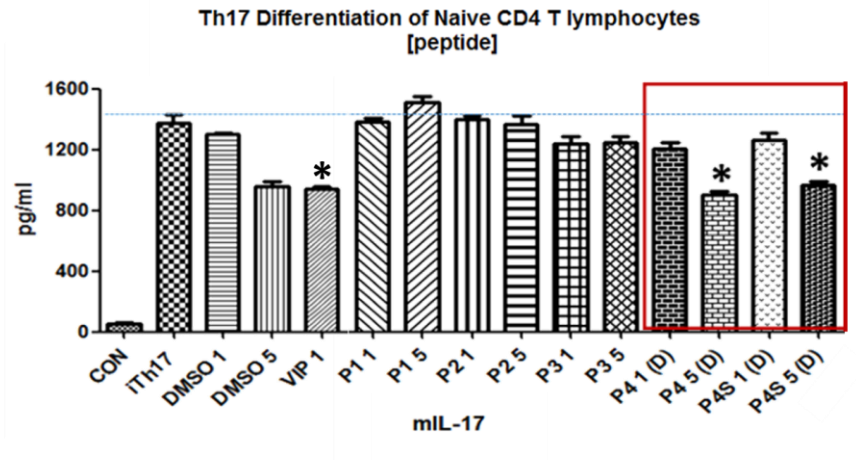

Supplementary 3. Anti-inflammatory test of tIK-YK1,2,3,4 and tIK-9 mer. Each is represented by P1,2,3,4,4S in order.
